# Measuring hedonia and eudaimonia as motives for activities: cross-national investigation through traditional and Bayesian structural equation modeling

**DOI:** 10.3389/fpsyg.2014.00984

**Published:** 2014-09-08

**Authors:** Aleksandra Bujacz, Joar Vittersø, Veronika Huta, Lukasz D. Kaczmarek

**Affiliations:** ^1^Faculty of Social Sciences, Institute of Psychology, Adam Mickiewicz UniversityPoznañ, Poland; ^2^Department of Psychology, University of TromsøTromsø, Norway; ^3^School of Psychology, University of OttawaOttawa, ON, Canada

**Keywords:** hedonia, eudaimonia, well-being, Bayesian structural equation modeling, measurement invariance

## Abstract

Two major goals of this paper were, first to examine the cross-cultural consistency of the factor structure of the Hedonic and Eudaimonic Motives for Activities (HEMA) scale, and second to illustrate the advantages of using Bayesian estimation for such an examination. Bayesian estimation allows for more flexibility in model specification by making it possible to replace exact zero constraints (e.g., no cross-loadings) with approximate zero constraints (e.g., small cross-loadings). The stability of the constructs measured by the HEMA scale was tested across two national samples (Polish and North American) using both traditional and Bayesian estimation. First, a three-factor model (with hedonic pleasure, hedonic comfort and eudaimonic factors) was confirmed in both samples. Second, a model representing the metric invariance was tested. A traditional approach with maximum likelihood estimation reported a misfit of the model, leading to the acceptance of only a partial metric invariance structure. Bayesian estimation—that allowed for small and sample specific cross-loadings—endorsed the metric invariance model. The scalar invariance was not supported, therefore the comparison between latent factor means was not possible. Both traditional and Bayesian procedures revealed a similar latent factor correlation pattern within each of the national groups. The results suggest that the connection between hedonic and eudaimonic motives depends on which of the two hedonic dimensions is considered. In both groups the association between the eudaimonic factor and the hedonic comfort factor was weaker than the correlation between the hedonic pleasure factor and the eudaimonic factor. In summary, this paper explained the cross-national stability of the three-factor structure of the HEMA scale. In addition, it showed that the Bayesian approach is more informative than the traditional one, because it allows for more flexibility in model specification.

## Introduction

The distinctions between hedonic and eudaimonic notions of happiness has attracted a rapidly expanding group of well-being researchers (e.g., Keyes et al., 2002; Kopperud and Vittersø, 2008; Berridge and Kringelbach, 2011; Henderson et al., 2013; Huta, 2013; Huta and Waterman, 2013; Oishi et al., 2013; Ryan et al., 2013; Bauer et al., 2014; Proctor et al., 2014; Uchida et al., 2014). Both concepts are derived from ancient philosophy and they came to prominence within positive psychology as a result of research that conceptualized well-being in different ways. Proponents of a strict hedonic approach argue that a good life is properly accounted for by the presence of pleasure and the absence of pain (Kahneman, 1999; Tännsjö, 2007), whereas a broader approach includes positive attitudes and life satisfaction within the idea of hedonia (e.g., Diener, 1984; Feldman, 2004; Diener et al., 2009). By contrast, proponents of eudaimonic approaches believe that there is more to a good life than pleasant feelings and favorable attitudes (Tatarkiewicz, 1976; Ryff, 1989; Waterman, 1993; Ryan and Deci, 2001; Deci and Ryan, 2008; Keyes and Annas, 2009; Vittersø, 2013). In the current study, we enter this debate by looking into an established self-report scale that measures both hedonic and eudaimonic conceptions of well-being—the Hedonic and Eudaimonic Motives for Activities scale (HEMA; Huta and Ryan, 2010). Our two major aims are, first to examine the cross-cultural consistency of the factor structure of the HEMA, and second to illustrate the advantages of using Bayesian estimation for such an examination.

Existing attempts to quantify the associations between elements of hedonic and eudaimonic well-being show mixed results. In a recent review, Huta and Waterman (2013) attributed some of these inconsistencies to conceptual disagreements. Four categories of conceptualizations were identified, as Huta and Waterman observed that well-being has interchangeably been studied as orientations, as behaviors, as experiences and as functioning. Another distinction in the well-being literature relates to the level of analyses: both trait level measures and state level measures were frequently observed. In order to avoid some of these confusions Huta and Waterman argued that it is important to clearly specify which category of analysis and level of measurement is taken into consideration. When focusing specifically on the distinction between eudaimonia and hedonia (as in a factor analysis) and on the stability of the correlation between them (as in a multigroup investigation) the confounding effect of different conceptualizations should be avoided. Therefore, the current paper operationalizes eudaimonia and hedonia as orientations, measured at the trait level. The HEMA scale fulfills both these criteria and was thus elected as the measurement instrument of our study.

The hedonic subscale of the HEMA addresses the two concepts that appear in most conceptions of hedonia: pleasure and absence of pain (e.g., Kahneman, 2000). The absence of pain is assessed in approach terms (as “seeking relaxation” and “seeking to take it easy”) rather than in avoidance terms to minimize the confounding role played by the differential effects of approach and avoidance motivation (for a review see Elliot, 2008). This corresponds with the argument that pleasure—a proactive search for positive experiences—should be distinguished from comfort defined as a state of biological indifference (Scitovsky, 1976; Cabanac, 2010). For this reason the hedonic scale could actually be divided into the two dimensions of seeking relaxation and seeking pleasure (Asano et al., 2014).

The eudaimonic subscale of the HEMA covers three concepts which focus on personal qualities: authenticity (self-knowledge, autonomy, and integrity), excellence (virtue, performing to a high standard), and growth (learning, actualizing one's unique potentials, and maturing as a person). This operationalization is not exhaustive, as other concepts are often emphasized in definitions of eudaimonia (e.g., meaning, engagement). Nevertheless, the advantage of the HEMA scale is that it allows for a simultaneous assessment of both hedonic and eudaimonic orientations (measured together as motives), and therefore provides an opportunity to study the connection between them.

The relationship between factors of stable hedonic and eudaimonic orientations to well-being is hardly ever studied across different samples or national groups. Therefore, we don't know whether a particular figure representing the correlation between the factors is universal or specific for a national sample or a language version. To confirm a stability of connections between well-being constructs multigroup studies are needed. Yet for such designs the issue of measurement invariance (MI) becomes a crucial concern (e.g., Brown, 2006). This means that when a measurement tool is used across groups, its internal structure should follow at least two requirements: (1) the same number of factors should occur in all groups (configural invariance), and (2) the similar pattern of factor loadings should be observed across groups (metric invariance). If a model that imposes both of those requirements fits the data well, structural parameters—such as factor correlations—can be legitimately examined and compared across groups (e.g., Meredith and Teresi, 2006; Raykov et al., 2012). Additionally, when a comparison between latent means is of interest, the similar pattern of item intercepts should be established (scalar invariance).

In sum, the aim of this paper is to provide a systematic investigation of the correlational nature of the HEMA scale in two different nations. A confirmatory and multigroup factor analytic design was chosen for this purpose.

## The application of bayesian estimation

With cross-national data from the HEMA scale, the analysis presented in this paper utilizes and compares two different estimation methods: (1) a traditional frequentist approach with maximum likelihood (ML) estimation and (2) a relatively new technique based on Bayesian structural equation models (BSEM) (Muthén and Asparouhov, 2012). This double analyses strategy was chosen in order to compare the results of those two methods and thereby provide an example that will reveal the possible advantages offered by Bayesian estimation. Since computational power nowadays supports the use of the Bayesian approach, it has been widely recommended due to the fundamental advantages of this method. Several introductory discussions of Bayesian estimation and inference exist (e.g., van de Schoot et al., 2013a; Zyphur and Oswald, 2013). The possible advantages of using BSEM can be found in all three steps of the analysis reported here.

First, BSEM allows the replacing of exact zero constraints with approximate zero constraints for different parameters of a model such as cross-loadings or residual covariances (Muthén and Asparouhov, 2012, 2013). This is possible due to the specific assumptions underlying Bayesian estimation. Bayesians treat parameters as variables characterized by a distribution, in contrast to the frequentist approach in which samples have distributions while parameters are fixed in the population (Zyphur and Oswald, 2013). Moreover, in Bayesian analysis a distribution for each of the parameters can be restricted by specifying priors, which are usually based on previous knowledge. For example, in confirmatory factor analyses (CFA) it is often assumed that each of the items will load on one factor only, hence the errant loadings (cross-loadings) are fixed at zero. However, the precise zero constraint has been criticized as unreasonable and unnecessary, because researchers usually want those errant-loadings to be very small (e.g., Golay et al., 2013). In most cases it may be enough to state that cross-loadings do not exceed a particular value, for example 0.3 (Brannick, 1995). Bayesian estimation allows us to place such a constraint by specifying a prior distribution for a cross-loading, in order to have little variance around the mean set to zero (i.e., an informative prior, e.g., van de Schoot et al., 2013b). Thanks to this option, the model fit will not suffer from an unreasonable assumption that does not reflect the true intention of the researcher.

Secondly, the same advantage of an approximate equality, rather than a precise one, can be employed for the MI analyses (Muthén and Asparouhov, 2013; Cieciuch et al., 2014). Traditional MI strategy places strong constraints on the parameters of a scale by forcing them to be identical across groups. Such an approach often leads to the conclusion that a scale is not invariant across groups, with little information about how big the differences are. Previous attempts to deal with this problem by establishing partial MI models remain controversial (e.g., Byrne et al., 1989; Millsap and Kwok, 2004; Schmitt and Kuljanin, 2008). In this study, as in many others, the goal is to show that a scale performs in a very similar way across national groups. Yet, it is not expected that any particular item will behave differently across the groups (this could be solved by a partial MI model). Instead, we assume that all the items in the scale may vary across the nations and the size of these differences is of interest. BSEM allows the estimation of their magnitude by specifying limits for their distribution (which are set up by the informative priors). Thus, we decided to employ an approximate MI approach based on BSEM, and assumed that small deviations (i.e., statistically insignificant) would not jeopardize the comparison between factor covariances.

Thirdly, Bayesian estimation makes the results easier to understand due to its intuitive inference process (van de Schoot et al., 2013a). In the frequentist approach, a confidence interval is provided which shows that over an infinity of samples taken from the population, 95% of these contain the true population value. The interpretation of such an interval is somewhat counterintuitive, as it refers to samples rather than an actual parameter of interest. On the other hand, a Bayesian credibility interval indicates that there is a 95% chance for a parameter to lay within the limits of the interval. Taking this paper as an example, the credibility interval will reflect the most probable range of values for the correlation between the latent factors reflecting hedonic and eudaimonic pursuits of well-being. In other words, Bayesian approach focuses on the magnitude of the parameter for a provided dataset. Such information, in contrast to the traditional confidence intervals, is easier to understand and compare between groups.

In sum, the paper provides a practical application of Bayesian estimation, and aims at investigating some differences between the traditional frequentist approach to CFA with that of a Bayesian approach.

## Materials and methods

### Participants

In the Polish sample, 386 adults were surveyed, of whom 79% were female. Their age ranged from 18 to 29 years (*M* = 21.26, *SD* = 1.75). The data collection was conducted in two waves: first (*n* = 197) with the full 9-item version of the scale, and second (*n* = 189) with the short 8-item version (see the Supplementary Material for the list of items included in both versions). The assessment was based on a structured, anonymous questionnaire investigating a number of lifestyle-related variables (see Kaczmarek et al., 2013). Participation was on a voluntary basis and administration took place during the respondents' free time.

The English sample consisted of 429 North American Anglophone participants. The study involved undergraduates (75% of women) who completed the questionnaire as part of a 1-h screening survey (including measures submitted by a variety of researchers) used as a preliminary step before granting students access to various individual studies. Their age ranged from 18 to 30 years (*M* = 19.19, *SD* = 1.92).

### Instrument

The HEMA scale is meant to assess motives for activities that can be divided into those that are eudaimonic (e.g., “seeking to develop the best in oneself”) and those that are hedonic (e.g., “seeking pleasure”). It is underlined by Huta and Ryan (2010)—the authors of the scale—that this approach allows the distinguishing of hedonia and eudaimonia as forms of well-being pursuits from well-being products. It also offers the opportunity to study both motives as separate variables. Thus, the HEMA measures eudaimonia and hedonia in parallel terms, operationalizing both as orientations (Huta and Ryan, 2010).

The HEMA is a 9-item instrument comprising a hedonic motivation subscale (5 items) and a eudaimonic motivation subscale (4 items). Responses range from 1 (not at all) to 7 (very much). A back-translation procedure was used to translate the HEMA scale into Polish by two bilingual psychologists (the original English items and their Polish translation are presented in the Supplementary Table 1). During this process, one of the items (“seeking enjoyment”), originally belonging to the hedonic subscale, was identified to have different cultural connotations. In the English language, it reflects striving after pleasant experiences, while in the Polish version, it may have been perceived as reflecting goal achievement, rather than being specific to hedonia or eudaimonia. Such disparity could be expected due to the existence of different well-being definitions across nations (e.g., Wierzbicka, 2004). In this situation a partial MI that leaves out the problematic item could have been employed. However, this would undermine the interpretation of estimated factor correlations (the item reflected the hedonic construct in the English version only). Therefore, in this article we tested a possibility to use the 8-item version of the scale in both language groups.

### Analysis

The analysis was conducted in three stages: (1) the dimensional structure of the scale was established through CFA separately for the two national samples, (2) the MI was tested between the countries, and finally (3) the differences between the latent factors' correlations were tested. The research question focuses on the construct validity of the scale, therefore a metric invariance (equality of factor loadings) was of main interest (Byrne, 2012). This type of MI indicates whether respondents across groups attribute the same meaning to the latent construct under the study (van de Schoot et al., 2012). In other words, indicators that are central to the construct in one national group, are also central in the other (Selig et al., 2008). It is assumed here that the similar pattern of item intercepts (scalar invariance) is not required for a meaningful comparison of factor covariances, even though this claim can be considered controversial by some researchers (for discussion see Byrne and van de Vijver, 2010). Although of secondary interest, further analyses of the scalar invariance (i.e., invariance of observed variables' intercepts) were also conducted, and differences between intercepts were tested. This allowed for a better illustration of the functioning of the scale in the two national groups.

All the analyses were performed using Mplus 7.11 (Muthén and Muthén, 1998–2012). For the traditional analyses, ML parameter estimates with standard errors and a chi-square test statistic robust to non-normality was used (MLR, see Muthén and Muthén, 1998–2012). When ML estimation was employed, for the evaluation of a model the following fit indices were used with the respective cut-off values as proposed by Schweizer (2010); χ^2^, normed χ^2^ (NC, with values below 3 indicating an acceptable fit and below 2 a good fit), CFI and TLI (acceptable model fit when higher than 0.90, good fit when higher than 0.95), RMSEA (acceptable fit when lower than 0.08, good fit when lower than 0.05) and SRMR (expected to stay below 0.10). Chi-square difference test (using the Satorra-Bentler scaled chi-square), AIC and BIC values were employed to compare models. In the Bayesian analyses, two indicators of a model fit were interpreted: (1) the posterior predictive *p*-value (PPP, good fit when equal to or higher than 0.05), and (2) the 95% confidence interval of the replicated chi-square value (which was expected to include zero; for details please refer to Muthén and Asparouhov, 2012). We additionally used the deviance information criterion to compare the model (DIC; Spiegelhalter et al., 2002). Model estimation was performed with maximum 500,000 and minimum 20,000 iterations using the Markov chain Monte Carlo (MCMC) algorithm (Muthén and Asparouhov, 2012). MCMC convergence criterion using potential scale reduction (PSR) was set to 0.01 (Gelman and Rubin, 1992). The alignment method for the approximate MI was not employed since it is not yet available for models with cross-loadings (Muthén and Asparouhov, 2014). The data and all Mplus output files are available in the Supplementary Materials.

## Results

### Factor structure

In the first step of the analysis a latent structure of the scale had to be established. Based on the theoretical assumptions underlying the HEMA scale, a model separating the hedonic and the eudaimonic factor was expected. Previous exploratory analyses revealed the existence of such two-factor structure (Huta and Ryan, 2010; Anić, 2014). However, a recent confirmatory analysis of the Japanese version of the HEMA scale revealed that a three-factor structure is a better representation of the scale's structure (Asano et al., 2014). Therefore, our goals were to (1) determine the factor structure of the scale, and (2) validate the performance of the short 8-item instrument. In order to do so, each national sample was divided into two groups. In the Polish sample the groups were formed according to the waves of the data collection (in the first wave the 9-item scale was administered, in the second wave the 8-item instrument was used). In the English sample participants were divided at random into two groups, and for the second group the “seeking enjoyment” item was removed from the analyses. Then, the one, two and three-factor solutions were tested in four samples. The analysis was begun with the traditional frequentist approach, followed by the Bayesian estimation.

The results acknowledged that the three-factor model was a better solution (see Table 1, syntaxes 1–7 included in the Supplementary Materials). In both national groups, and for both the short and full versions of the scale, splitting the hedonic factor into two components would notably improve the fit. Due to both the theoretical and empirical plausibility of such a distinction, we decided to continue the analyses with the three-factor structure. The proposed three-factor model categorized the hedonic items into a comfort (“seeking to take it easy”; “seeking relaxation”) and a pleasure group (“seeking fun”; “seeking pleasure”; “seeking enjoyment” in the full version of the scale). The confirmatory procedure verified this model by revealing its acceptable fit in both the English and Polish samples (Table 1). In this traditional approach to the CFA, no cross-loadings between items were allowed.

**Table 1:** **The confirmatory factor analyses using maximum likelihood estimation with robust standard errors (ML)**.

	**NC**	**χ^2^**	***df***	***p***	**RMSEA**	**CFI**	**TLI**	**SRMR**	**AIC**	**BIC**
**ENGLISH**										
**9-items (*N* = 223)**										
1 factor	12.09	326.50	27	<0.001	0.22	0.55	0.40	0.15	6634	6726
2 factors	5.49	142.67	26	<0.001	0.14	0.82	0.76	0.09	6430	6526
3 factors	2.13	55.44	26	<0.001	0.08	0.95	0.93	0.05	6334	6436
**8-items (*N* = 206)**										
1 factor	8.56	171.15	20	<0.001	0.19	0.60	0.45	0.13	5682	5762
2 factors	4.86	92.27	19	<0.001	0.14	0.81	0.72	0.09	5581	5664
3 factors	1.84	31.36	17	0.02	0.06	0.96	0.94	0.05	5507	5597
**8-items, full sample (*N* = 429)**										
3 factors	3.36	57.16	17	<0.001	0.07	0.95	0.92	0.05	11,303	11,412
**POLISH**										
**9-items (*N* = 197)**										
1 factor	4.25	114.90	27	<0.001	0.13	0.81	0.74	0.10	4967	5056
2 factors	2.39	62.17	26	<0.001	0.08	0.92	0.89	0.07	4907	4999
3 factors	1.74	41.82	24	0.01	0.06	0.96	0.94	0.06	4889	4987
**8-items (*N* = 189)**										
1 factor	6.47	129.46	20	<0.001	0.17	0.65	0.51	0.12	4525	4603
2 factors	3.46	65.73	19	<0.001	0.11	0.85	0.78	0.07	4451	4532
3 factors	2.39	40.76	17	0.001	0.09	0.92	0.87	0.06	4427	4515
**8-items, full sample (*N* = 386)**										
3 factors	2.50	42.60	17	<0.001	0.06	0.96	0.94	0.05	8878	8985

In terms of the short vs. the full version of the scale, the results were somewhat inconclusive. In the English sample the short version (with one item excluded from the analysis) fitted the data slightly better. In the Polish sample, however, the fit was worse when the 8-item version of the scale was administered. To check whether these differences represented the specific variability of a group, rather than a general tendency, we have retested the chosen three-factor model on the full English and Polish samples (using the short version of the scale). This resulted with acceptable fit in both national samples leading to a conclusion that the 8-item instrument produces similar factor structure to the one detected for the full version of the scale.

#### Bayesian CFA

The factor structure of the HEMA scale was then re-tested using Bayesian estimation (see Table 2 for results, and syntaxes 8–11). First, the noninformative prior distribution was specified using the default prior settings available in Mplus (Muthén and Muthén, 1998–2012). Therefore, no previous knowledge was imposed, meaning that every value of a parameter was equally likely to occur (van de Schoot et al., 2013a). In this case, cross-loadings were fixed to zero, just as in the ML estimated models. With this specification, the two-factor and three-factor models were tested. The one-factor model was omitted for clarity, and due to its very poor fit as revealed in the previous analysis.

**Table 2:** **The confirmatory factor analyses using Bayesian estimation**.

	**#fp**	**2.5% pp**	**97.5% pp**	**PPP**	**DIC**
**ENGLISH**					
**9-items (*N* = 223)**					
2 factors NI	28	107.09	157.27	<0.01	6432
2 factors CL	37	92.71	147.25	<0.01	6424
3 factors NI	30	6.35	60.21	<0.01	6335
3 factors CL	48	−20.42	34.94	0.34	6314
**8-items (*N* = 206)**					
2 factors NI	25	74.39	123.23	<0.01	5582
2 factors CL	33	49.83	107.29	<0.01	5564
3 factors NI	27	−0.25	47.65	0.03	5509
3 factors CL	43	−14.84	39.37	0.17	5501
**8-items, full sample (*N* = 429)**					
3 factors CL	43	−16.71	35.67	0.23	11,265
**POLISH**					
**9-items (*N* = 197)**					
2 factors NI	28	19.54	70.05	<0.01	4909
2 factors CL	37	−20.30	37.21	0.28	4877
3 factors NI	30	0.27	55.22	0.02	4895
3 factors CL	48	−24.75	33.42	0.38	4875
**8-items (*N* = 189)**					
2 factors NI	25	28.94	78.39	<0.01	4451
2 factors CL	33	19.75	72.00	<0.01	4447
3 factors NI	27	6.25	53.19	<0.01	4430
3 factors CL	43	−3.61	54.19	0.04	4427
**8-items, full sample (*N* = 386)**					
3 factors CL	43	−12.27	37.86	0.14	8864

*NI, Noninformative priors; CL, Informative priors on cross-loadings have a zero mean and a variance of 0.01*.

In almost all situations neither the two- nor three-factor model resulted in a satisfactory fit (i.e., the PPP was significant, and the 95% CI of the replicated chi-square values did not include zero). The only exception was the 8-item scale in the English group, where the three-factor model resulted in close to acceptable fit (i.e., the PPP was significant, but the 95% CI included zero). We then changed the requirements of the model so that cross-loadings would be approximately zero rather than exactly zero (syntaxes 12–16). Using small-variance priors (prior mean = 0; prior variance = 0.01) all cross-loadings were restricted to having a value ranging from −0.2 to 0.2 (for more choices please refer to Muthén and Asparouhov, 2012, p. 316). The goal of this strategy was to allow for cross-loadings, yet at the same time keep them small and statistically insignificant. This resulted in an improvement of the three-factor model fit, yet did not help in the case of the two-factor model (see Table 2). Thus, it was again concluded that the three-factor model fit the data better and the analyses were continued employing this structure.

Thanks to the use of weakly informative priors for cross-loadings the results of the Bayesian CFA provided some interesting insights into the performance of the short and full versions of the scale. In the English sample both the 9- and 8-item versions resulted with a good fit when cross-loadings were introduced in the three-factor solution (PPP was accordingly 0.34 and 0.17). In the Polish sample the short version of the scale responded with improvement into an almost acceptable model fit (PPP = 0.04; 95% CI included zero). However, when cross-loadings were allowed in the full version the model yielded a satisfactory fit also for the two-factor solution (PPP = 0.28). In both two- and three-factor models cross-loadings for the problematic “seeking enjoyment” item were large enough to become significant (see the Supplementary Table 3 for details). This suggested that in the Polish sample the 8-item version of the scale represents the measured constructs in a more clear way. Finally, the analyses conducted on the full English and Polish samples confirmed the fit of the three-factor model with small cross-loadings.

### Measurement invariance

In the second step of the analysis, a series of multi-group CFA were executed in order to test the MI between the Polish and the English versions of the HEMA scale (syntaxes 17–21). Stepwise procedures were employed, where the analysis begins with the least restricted solution and subsequent models with increasingly restrictive constraints are evaluated (Brown, 2006). Comparisons were performed with a corrected chi-square differences test due to the fact that the analyses were based on a robust maximum likelihood method (MLR; Muthén and Muthén, 1998–2012). In order to identify the model the factor variance was fixed to 1 in one group only, and in the other group the equality constraints were placed on the factor loadings while a factor variance was estimated (Yoon and Millsap, 2007). This method minimizes problems caused by commonly used solutions such as constraining the first factor loading to one (Bauer and Hussong, 2009).

With a configural invariance model (syntax 17) established across the two language versions of the HEMA, the next step was to test for metric invariance (see Table 3). A model constraining factor loadings to being equivalent across the language versions (syntax 18) fitted slightly worse than a configural model. Then a partial metric invariance model (syntax 19) was tested, where one factor loading was allowed to vary across groups (“seeking relaxation”). This specification represented the data well. Accordingly, the construct validity of the scale was confirmed across the national samples enabling a meaningful comparison between the factor covariances. Finally, a scalar invariance was tested (syntax 20). The results were not supportive for the scalar invariance indicating that the intercepts were not equal across the samples. Partial scalar model (syntax 21) could have been established only when half of the intercepts were allowed to vary. We have therefore retreated to the partial metric MI model and this one was applied for the final step of the analyses (see **Table 5** for standardized results of the partial metric MI model).

**Table 3:** **The measurement invariance analyses using ML**.

	**NC**	**χ^2^**	***df***	**RMSEA**	**CFI**	**TLI**	**SRMR**	**Δχ^2 adj^**	**Δ*df***	**Δ*p***
Configural	2.95	100.19	34	0.07	0.96	0.93	0.05	−	−	−
Metric	2.92	113.73	39	0.07	0.95	0.93	0.06	13.61	5	0.02
Partial^a^	2.78	105.64	38	0.07	0.95	0.93	0.06	5.98	4	0.20
Scalar	5.55	238.86	43	0.11	0.87	0.83	0.08	179.32	5	<0.001
Partial^b^	2.75	107.327	39	0.07	0.95	0.93	0.06	1.38	1	0.24

a *Free factor loading of item 1 (relaxation), the partial metric model is compared to the configural model*.

b *Free intercepts of items 4 (pleasure), 3 (do what you believe), 2 (learn, develop skills), and 1 (relaxation), the partial scalar model is compared to the partial metric model*.

#### Approximate MI

Then, the MI across groups was re-tested with the Bayesian estimator (see Table 4). We have continued with the model where weakly informative priors on the cross-loadings were used (i.e. small cross-loadings were allowed, see syntaxes 22–26). The configural model (syntax 22) fitted the data well as expected, given the previously confirmed stability of the three-factor model across the national groups. The full metric model (syntax 23) resulted in an acceptable fit, yet the PPP-value was still quite low (0.057). We have proceeded to establish and approximate metric invariance model (syntax 24), resulting in a slightly better fit (higher PPP-value, 0.078), but the difference in DIC was small (equals 2.1). Therefore, we have decided to employ the metric invariance model, not the approximate one, in the further analysis. The standardized factor loadings and intercepts estimated for this model are presented in Table 5. Lastly, the scalar invariance (syntax 25) was tested, but the model did not represent the data well. Therefore, the strict equality assumption between the intercepts was released, and the approximate scalar MI (syntax 26) was implemented (Muthén and Asparouhov, 2013). Allowing all the intercepts to be at least approximately equal (prior mean = 0; prior variance = 0.01) did not help to improve the fit. In fact neither of the methods used (the partial invariance with ML or the approximate invariance with Bayes) supported scalar invariance. This suggests that the problem of non-invariant intercepts is not limited to a particular item(s), and that all the items vary to an extent that cannot be disregarded (only for the item 5 credibility intervals for the intercepts overlapped) The intercepts in the Polish sample were higher than the ones of the English sample (see Table 5). Thus, the comparison of factor means would not be possible. Yet, in order to compare factor covariances the metric model could be used (Byrne and van de Vijver, 2010).

**Table 4:** **The measurement invariance analyses using Bayesian estimation**.

**BSEM**	**#fp**	**2.5% pp**	**97.5% pp**	**PPP**	**DIC**
Configural	86	−13.57	61.47	0.102	20,133
Metric	81	−10.53	67.75	0.057	20,131
Metric approximate^a^	89	−10.97	60.773	0.078	20,129
Scalar	76	21.63	91.06	0.001	20,154
Scalar approximate^a^	84	11.76	86.44	0.005	20,149

a *Informative priors on differences between groups have a zero mean and a variance of 0.01*.

**Table 5:** **Standardized factor loadings and intercepts for the metric invariance model**.

	ML				Bayes			
	Polish		English		Polish		English	
	**FL**	**Int**	**FL**	**Int**	**FL**	**Int**	**FL**	**Int**
**HEDONIC PLEASURE**								
Item 4 (pleasure)	0.82	5.31	0.80	3.52	0.76	5.28	0.73	3.51
Item 8 (fun)	0.68	3.94	0.80	3.64	0.74	3.88	0.86	3.65
**HEDONIC COMFORT**								
Item 1 (relaxation)	0.93	4.46	0.70	2.70	0.88	4.40	0.82	2.72
Item 6 (easy)	0.75	4.32	0.90	2.65	0.81	4.32	0.77	2.63
**EUDAIMONIC FACTOR**								
Item 2 (learn, develop skills)	0.50	5.37	0.62	3.77	0.55	5.35	0.67	3.74
Item 3 (do what you believe)	0.51	4.81	0.61	3.05	0.51	4.81	0.60	3.02
Item 5 (pursue excellence)	0.57	4.58	0.80	3.80	0.59	4.52	0.82	3.80
Item 7 (use the best in yourself)	0.60	4.83	0.80	3.74	0.56	4.78	0.74	3.74

*FL, Factor loadings; Int, Intercepts. Partial metric invariance model in the ML estimation. Cross-loadings omitted for clarity*.

### Correlation between hedonia and eudaimonia

The third and final step of the analysis was to quantify and compare the covariances between the latent factors representing hedonic and eudaimonic pursuits of well-being (syntaxes 27–28). To do so, the MODEL CONSTRAINT command was included in the Mplus code (Muthén and Muthén, 1998–2012). This function allowed us to create a set of new parameters representing the differences between the estimated factor covariances across and within the national groups. Mplus provided us with confidence intervals (using the Delta method standard errors and *z*-test for ML estimation) or credibility intervals (for Bayesian estimation) for the newly defined parameters. As a result, the differences between the factor covariances were tested for statistical significance. We continued to use partial metric invariance model for MI estimation (syntax 27), and metric invariance model with small cross-loadings specified with weakly informative priors for Bayes (syntax 28).

Table 6 presents the factor correlations (standardized covariances) for the two national groups and the two estimation methods, marked for the significant differences between and within groups. Both the traditional and Bayesian procedures revealed similar latent factor correlation patterns within each of the national groups. In the Polish sample the connection between the two hedonic factors was found to be the strongest (significantly stronger than each of the other two correlations), while the correlations between the hedonic factors and the eudaimonic factor were rather weak. In the English sample the links connecting the hedonic factors, and the hedonic pleasure with the eudaimonic factor were moderate. The correlation between the hedonic comfort and the eudaimonic factor was weak and mostly insignificant (only in the Polish group with ML *p* = 0.02). It was weaker than each of the other two correlations in all cases except from ML estimation in the English group. Between group differences were found in the connection between hedonic comfort with the eudaimonic factor (with ML) or in the hedonic pleasure with eudaimonic factor (with Bayes). Even though the correlation between the hedonic factors was stronger in the Polish group than the English group, neither ML nor Bayes found this difference significant.

**Table 6:** **Correlations between latent factors of hedonia and eudaimonia as estimated with ML and Bayes**.

	ML (95% *CI*)		Bayes (BCI)	
	**Polish**	**English**	**Polish**	**English**
Hedonic pleasure with hedonic comfort	0.82^1^ (0.74; 0.91)	0.46^1^ (0.32; 0.59)	0.82^1^ (0.72; 0.90)	0.47^1^ (0.28; 0.62)
Hedonic pleasure with eudaimonic	0.29^2^ (0.12; 0.46)	0.54^1^ (0.42; 0.67)	0.26^2a^ (0.01; 0.48)	0.50^1a^ (0.31; 0.66)
Hedonic comfort with eudaimonic	0.18^2a^ (0.03; 0.33)	0.09^1a^ (-0.01; 0.25)	0.17^2^ (-0.06; 0.38)	0.16^2^ (-0.03; 0.34)

*Correlations marked with superscript letter “a” differ between national groups. Correlations within one column not sharing the same superscript number differ within national groups. CI, Confidence interval; BCI, Bayesian credibility interval*.

## Discussion

The aim of this paper was to describe the structure of the HEMA scale and its performance across two different nations. Stepwise analyses were conducted to establish a factor structure of the scale, revealing three correlated factors: two hedonic and one eudaimonic. The eudaimonic factor reflected the pursuit for excellence. The hedonic factors include items reflecting the pursuit of affective states and were divided into a comfort factor and a pleasure factor.

Among the two hedonic factors, the one reflecting pleasure was closer to the eudaimonic factor than was the hedonic comfort factor. This pattern was relatively stable across the national groups. Thus, seeking excellence seems to feel more like pleasure and fun, than like being relaxed and at ease. In fact, some researchers include the enjoyment from activities representing the pursuit of excellence into their definition of eudaimonia (Waterman et al., 2010). At the same time, both the hedonic factors were strongly correlated, indicating that these items roughly occupy the same area of the affective landscape. Splitting hedonia into the two components shed more light on unclear previous results regarding its connection to eudaimonia (Huta and Waterman, 2013). This division may be valid for further research, yet building a dedicated scale to assess hedonic comfort and pleasure is recommended.

This paper is a first attempt at a systematic examination of multigroup stability of the HEMA scale components. Future work should address several issues not answered in this study. First of all, the conclusions of this paper are based on the models that fit acceptably well, yet are not perfect. More national samples should be taken into consideration to further justify the cross-national stability of a well-being assessment, including both hedonic and eudaimonic constructs. Secondly, representative samples are preferred in order to avoid possible sampling errors. Especially the lack of gender balance is an important limitation of this study. Thirdly, the lack of scalar invariance revealed in this study should be examined more closely. The differences between intercepts are not surprising when comparing across nations, as they might occur as a result of different norms of socially-acceptable levels for expressions of hedonia and eudaimonia (e.g., Diener, 2000). Detailed analyses of this phenomenon were not within the scope of this paper, but remain an interesting issue. Finally, the division of hedonic comfort and pleasure needs further attention, and a more detailed assessment of those hedonic components could be considered.

In summary, this paper revealed a similar pattern of correlations between the trait-level pursuits of hedonic and eudaimonic elements across the two national samples. Further cross-national studies are needed to confirm the existence of this pattern, as well as to explain the differences in the items' intercepts across the groups (the lack of scalar invariance). It is hoped that using the HEMA scale in various language versions and across different national groups has the potential to substantially advance the knowledge on hedonic and eudaimonic components of well-being.

## The performance of the bayesian estimation

Bayesian estimation was employed in this study due to its fundamental advantages over the traditional frequentist approach (e.g., Muthén and Asparouhov, 2012; van de Schoot et al., 2013a; Zyphur and Oswald, 2013). It was expected that specifying weakly informative priors would help us to better assess the differences between groups, and the intuitive inference process would provide a simpler interpretation of the factor correlations. Several points regarding the fulfillment of those expectations are discussed here.

Firstly, Bayesian estimation reported a misfit of the model when strong assumptions of exact zero were imposed. This is interesting given that the estimation based on the ML method reported an acceptable fit. The reason for this lies within the definition of a model fit used in Bayesian estimation. The posterior predictive checking assesses how well a model is specified from the viewpoint of predictive accuracy (how well it predicts the data). Thus, any discrepancies are detected between the values generated by a model and the observed data, suggesting that the model could be improved (van de Schoot et al., 2013a).

Consequently, in Bayesian estimation replacing the exact zero assumption with an approximate zero improved the fit significantly, leading to the acceptance of the model with small cross-loadings. In fact Bayes arrived at a similar outcome to that of the traditional estimation, yet using a longer route. This detour, however, was much more informative. While in the ML approach the CFA is not able to provide information about the reason of model misfit, Bayesian modeling gives more hints about it. Including small priors for cross-loadings (or residual covariances which is also possible, see Muthén and Asparouhov, 2012) helps in verifying why the model does not represent the data well. Yet, it should be underlined that when large discrepancies were observed, such as when scalar invariance was imposed, changing the exact zero assumption into the approximate one (in this case by specifying an approximate MI) did not help in achieving a satisfactory fit. Clearly, according to both ML and Bayes, the differences between the items' intercepts were too big for scalar invariance to be established. This shows that the Bayesian approach can be more informative than ML only when the models are already fairly well specified. Indeed, this method is advised for analyses with a small number of groups, continuous variables and close-to-invariant models (van de Schoot et al., 2013b).

Finally, taking into account the small cross-loadings might have been the reason why the Bayesian estimation did not discover any differences between factor loadings (allowing for full metric invariance). Interestingly, for both methods the estimated factor correlations were almost identical, even though ML used only the partial metric MI model. In this case including small cross-loadings did not influence the structural parameters of the model. In fact, it helped in establishing the metric invariance. This might suggest that non-invariant cross-loadings (not included in the traditionally estimated metric MI model) could actually be the reason for its misfit. Such possibility opens up an interesting discussion, but simulation studies are needed to better understand the role of small and sample specific cross-loadings in multigroup MI analyses.

To summarize, Bayesian estimation can be a recommended approach to MI analyses when (1) small differences between groups are expected and the size of those differences should be estimated, and (2) when structural parameters are of interest (e.g., factor covariances) and a researcher would like to be provided with easy to interpret credibility intervals for such parameters.

### Conflict of interest statement

The authors declare that the research was conducted in the absence of any commercial or financial relationships that could be construed as a potential conflict of interest.
