# Supplementary material for: Measuring hedonia and eudaimonia as motives for activities: cross-national investigation through traditional and Bayesian structural equation modeling
Source: Front Psychol. 2014 Sep 8;5:984. doi: 10.3389/fpsyg.2014.00984 (PMC4157462; doi:10.3389/fpsyg.2014.00984)
Supplement: Supplementary file 2 [file DataSheet2.DOCX]

***Supplementary Material***

**Measuring hedonia and eudaimonia as motives for activities: Cross-national investigation through traditional and Bayesian structural equation modeling**

**Aleksandra Bujacz^1,2^*, Joar Vittersø^2^, Veronika Huta^3^, Lukasz Dominik Kaczmarek^1^**

^1^Institute of Psychology, Faculty of Social Sciences, Adam Mickiewicz University, Poznań, Poland

^2^Department of Psychology, University of Tromsø, Tromsø, Norway

^3^School of Psychology, University of Ottawa, Ottawa, Canada

*** Correspondence:** Aleksandra Bujacz, Institute of Psychology, Faculty of Social Sciences, Adam Mickiewicz University, ul. Szamarzewskiego 89AB, Poznań, 60-568, Poland.

aleksandra.bujacz@amu.edu.pl

1. **Article highlights**

- Investigation into the strength of connections between hedonic and eudaimonic motives for activities.
- Determining the cross-national stability of HEMA scale through measurement invariance analysis.
- An empirical example of applying Bayesian estimation, in comparison to the frequentist approach.

1. **The HEMA scale**

**Supplementary Table 1. The Items of the HEMA Scale in the Original (English) and the Translated (Polish) Version.**

| **Item** | **English version** | **Polish version** |
| --- | --- | --- |
| 1 (H) | Seeking relaxation | Dążenie do odprężenia |
| 2 (E) | Seeking to develop a skill, learn, or gain insight into something | Dążenie do rozwoju umiejętności, uczenia się lub zrozumienia czegoś |
| 3 (E) | Seeking to do what you believe | Dążenie do robienia tego, w co wierzysz |
| 4 (H) | Seeking pleasure | Dążenie do przyjemności |
| 5 (E) | Seeking to pursue excellence or a personal ideal | Dążenie do doskonałości lub osobistego ideału |
| 6 (H) | Seeking to take it easy | Dążenie do relaksu |
| 7 (E) | Seeking to use the best in yourself | Dążenie do wykorzystywania tego, co w tobie najlepsze |
| 8 (H) | Seeking fun | Dążenie do zabawy |
| Excluded | Seeking enjoyment | Dążenie do zadowolenia |

*Note.* H = Hedonic item; E = Eudaimonic item. English version reprinted from Huta, V., and Ryan, R. M. (2010). Pursuing pleasure or virtue: The differential and overlapping well-being benefits of hedonic and eudaimonic motives. *J. Happiness Stud. 11*, 735–762. doi:10.1007/s10902-009-9171-4

1. **Standardized factor loadings and correlations between factors in the tested models using ML and Bayes**

**Supplementary Table 2. Standardized factor loadings and correlations between factors in the tested models using ML.**

|  | **Polish** | | | | | | **English** | | | | | |
| --- | --- | --- | --- | --- | --- | --- | --- | --- | --- | --- | --- | --- |
|  | 9-items (*N*=197) | | | 8-items (*N*=189) | | | 9-items (*N*=223) | | | 8-items (*N*=206) | | |
|  | 1F | 2F | 3F | 1F | 2F | 3F | 1F | 2F | 3F | 1F | 2F | 3F |
| *Factor loadings* |  |  |  |  |  |  |  |  |  |  |  |  |
| Item 1 (relaxation) | **.87** | **.89** | **.94** | **.89** | **.89** | **.94** | **.34** | **.33** | **.80** | **.27** | **.44** | **.65** |
| Item 2 (learn, develop skills) | .07 | **.48** | **.48** | .00 | **.46** | **.45** | **.40** | **.71** | **.70** | **.54** | **.58** | **.57** |
| Item 3 (do what you believe) | .12 | **.49** | **.47** | .01 | **.37** | **.37** | **.43** | **.64** | **.64** | **.64** | **.65** | **.64** |
| Item 4 (pleasure) | **.79** | **.79** | **.79** | **.67** | **.67** | **.83** | **.86** | **.86** | **.86** | **.56** | **.70** | **.73** |
| Item 5 (pursue excellence) | .17 | **.64** | **.66** | .12 | **.65** | **.67** | **.45** | **.80** | **.80** | **.74** | **.76** | **.76** |
| Item 6 (easy) | **.72** | **.73** | **.73** | **.77** | **.77** | **.76** | **.30** | **.31** | **.79** | .21 | **.50** | **.96** |
| Item 7 (use the best in yourself) | **.37** | **.55** | **.55** | **.26** | **.68** | **.66** | **.52** | **.77** | **.77** | **.78** | **.82** | **.83** |
| Item 8 (fun) | **.66** | **.65** | **.66** | **.58** | **.58** | **.69** | **.78** | **.80** | **.80** | **.53** | **.85** | **.84** |
| Excluded item (enjoyment) | **.70** | **.67** | **.73** |  |  |  | **.90** | **.92** | **.92** |  |  |  |
| *Factor correlation* |  |  |  |  |  |  |  |  |  |  |  |  |
| Hedonia with Eudaimonia |  | **.30** |  |  | .21 |  |  | **.51** |  |  | **.51** |  |
| Comfort with Pleasure |  |  | **.90** |  |  | **.74** |  |  | **.38** |  |  | **.49** |
| Pleasure with Eudaimonia |  |  | **.44** |  |  | **.28** |  |  | **.51** |  |  | **.56** |
| Comfort with Eudaimonia |  |  | .18 |  |  | .17 |  |  | .13 |  |  | .08 |

*Note.* 1F = 1 factor; 2F= 2 factors; 3F = 3 factors; 3FF = 3 factors tested on the full sample. All factor loadings significant. Significant correlations marked in bold.

**Supplementary Table 3. Standardized factor loadings and correlations between factors in the tested models using Bayes.**

|  | **Polish** | | | | | | | | **English** | | | | | | | |
| --- | --- | --- | --- | --- | --- | --- | --- | --- | --- | --- | --- | --- | --- | --- | --- | --- |
|  | 9-items (*N*=197) | | | | 8-items (*N*=189) | | | | 9-items (*N*=223) | | | | 8-items (*N*=206) | | | |
|  | 2NI | 2CL | 3NI | 3CL | 2NI | 2CL | 3NI | 3CL | 2NI | 2CL | 3NI | 3CL | 2NI | 2CL | 3NI | 3CL |
| *Factor loadings* |  |  |  |  |  |  |  |  |  |  |  |  |  |  |  |  |
| Item 1 (relaxation) | **.89** | **.92** | **.93** | **.93** | **.89** | **.90** | **.92** | **.95** | **.34** | **.34** | **.79** | **.77** | **.44** | **.55** | **.68** | **.67** |
| Item 2 (learn, develop skills) | **.48** | **.52** | **.47** | **.53** | **.45** | **.48** | **.45** | **.50** | **.70** | **.72** | **.70** | **.73** | **.58** | **.62** | **.57** | **.64** |
| Item 3 (do what you believe) | **.48** | **.48** | **.46** | **.48** | **.37** | **.37** | **.36** | **.38** | **.64** | **.62** | **.64** | **.63** | **.65** | **.63** | **.64** | **.64** |
| Item 4 (pleasure) | **.79** | **.77** | **.79** | **.73** | **.67** | **.65** | **.82** | **.76** | **.86** | **.82** | **.85** | **.82** | **.70** | **.59** | **.73** | **.71** |
| Item 5 (pursue excellence) | **.63** | **.67** | **.65** | **.68** | **.65** | **.68** | **.66** | **.70** | **.80** | **.84** | **.80** | **.83** | **.76** | **.75** | **.75** | **.75** |
| Item 6 (easy) | **.73** | **74** | **.74** | **.73** | **.77** | **.77** | **.77** | **.75** | **.31** | **.35** | **.80** | **.84** | **.50** | **.66** | **.92** | **.93** |
| Item 7 (use the best in yourself) | **.55** | **.49** | **.55** | **.48** | **.68** | **.63** | **.67** | **.61** | **.77** | **.73** | **.77** | **.73** | **.82** | **.80** | **.82** | **.79** |
| Item 8 (fun) | **.66** | **.64** | **.65** | **.64** | **.58** | **.58** | **.68** | **.72** | **.80** | **.84** | **.80** | **.81** | **.85** | **.77** | **.84** | **.86** |
| Excluded item (enjoyment) | **.69** | **.63** | **.72** | **.68** |  |  |  |  | **.92** | **.90** | **.92** | **.95** |  |  |  |  |
| *Factor correlation* |  |  |  |  |  |  |  |  |  |  |  |  |  |  |  |  |
| Hedonia with Eudaimonia | **.30** | **.28** |  |  | **.21** | .20 |  |  | **.51** | **.49** |  |  | **.51** | **.43** |  |  |
| Comfort with Pleasure |  |  | **.93** | **.97** |  |  | **.75** | **.74** |  |  | **.36** | **.38** |  |  | **.51** | **.48** |
| Pleasure with Eudaimonia |  |  | **.43** | **.36** |  |  | **.23** | .30 |  |  | **.51** | **.51** |  |  | **.56** | **.54** |
| Comfort with Eudaimonia |  |  | .20 | .20 |  |  | .17 | .17 |  |  | .12 | .14 |  |  | .11 | .12 |
| *Cross-loadings* |  |  |  |  |  |  |  |  |  |  |  |  |  |  |  |  |
| Item 1 (relaxation) |  | -.08 |  | .03 |  | -.03 |  | .00 |  | .00 |  | .05 |  | -.03 |  | .06 |
| Item 2 (learn, develop skills) |  | -.07 |  | -.04 |  | -.08 |  | -.09 |  | -.04 |  | -.05 |  | -.07 |  | -.09 |
| Item 3 (do what you believe) |  | -.03 |  | -.02 |  | -.04 |  | -.04 |  | .03 |  | .08 |  | .02 |  | .03 |
| Item 4 (pleasure) |  | .03 |  | -.02 |  | .08 |  | .06 |  | .07 |  | .06 |  | **.12** |  | .05 |
| Item 5 (pursue excellence) |  | -.03 |  | -.02 |  | -.02 |  | -.03 |  | -.04 |  | -.05 |  | .01 |  | .02 |
| Item 6 (easy) |  | -.05 |  | -.01 |  | -.03 |  | .00 |  | -.06 |  | -.05 |  | **-.11** |  | .01 |
| Item 7 (use the best in yourself) |  | **.15** |  | .09 |  | .08 |  | .07 |  | .06 |  | .08 |  | .02 |  | .05 |
| Item 8 (fun) |  | .01 |  | .02 |  | .01 |  | -.04 |  | -.06 |  | .06 |  | .06 |  | .03 |
| Excluded item (enjoyment) |  | **.23** |  | **.18** |  |  |  |  |  | .03 |  | -.04 |  |  |  |  |

*Note.* 2 = two factors; 3 = three factors; NI = Noninformative priors; CL = Cross-loadings (prior .01). Significant factor loadings and correlations marked in bold. For the three-factor models the strongest cross-loading is presented.
